# The Predictive Role of Maternal Serum Amyloid A in Preterm Birth: An Observational Study in Romania

**DOI:** 10.7759/cureus.74996

**Published:** 2024-12-02

**Authors:** Evelina Chiriac, Zoran L Popa, Florin I Gorun, Narcis Vilceanu, Razvan Oros, Liana-Camelia Buhas, Patrick Dumitrescu, Cosmin Citu, Katalin Midia Tivadar, Andrei Csep, Bogdan Adrian Buhas

**Affiliations:** 1 Doctoral School, Faculty of Medicine and Pharmacy, University of Oradea, Oradea, ROU; 2 Department of Obstetrics-Gynecology and Neonatology, Faculty of Medicine, "Victor Babeş" University of Medicine and Pharmacy, Timisoara, ROU; 3 Department of Obstetrics and Gynecology, Timisoara Municipal Emergency Clinical Hospital, Timisoara, ROU; 4 Department of Morphological Disciplines, Faculty of Medicine and Pharmacy, University of Oradea, Oradea, ROU; 5 Department of Psycho-Neuroscience and Recovery, Faculty of Medicine and Pharmacy, University of Oradea, Oradea, ROU; 6 General Medicine School, Faculty of Medicine, "Victor Babeş" University of Medicine and Pharmacy, Timisoara, ROU; 7 Department of Urology, Faculty of Medicine and Pharmacy, University of Oradea, Oradea, ROU

**Keywords:** inflammatory biomarkers, maternal inflammation, predictive marker, preterm birth, serum amyloid a

## Abstract

Background: Despite improvements in pregnancy care, preterm birth remains a major cause of neonatal morbidity and mortality worldwide, particularly in developing countries. Maternal inflammation has been recognized as a factor that may induce preterm birth, with various inflammatory markers associated with its pathogenesis. The aim of this study is to evaluate the value of maternal serum amyloid A(SAA) level as a predictive marker for preterm delivery in a Romanian cohort.

Methods: This observational study was carried out at a Romanian tertiary care hospital between April 2023 and March 2024. We enrolled 136 pregnant women and divided them into two groups depending on the beginning of labor: preterm (before 37 weeks, n=70) and term (after 37 weeks, n=66). Maternal blood samples were taken upon admission and analyzed using the Atellica® NEPH 630 System (Siemens Healthineers, Erlangen, Germany) to determine SAA levels. The best cut-off value for SAA was determined using receiver operating characteristic (ROC) curve analysis with the Youden index. Logistic regression models were then applied to assess the association between elevated SAA levels and preterm birth, adjusting for potential confounders such as maternal age and history of preterm birth.

Results: The median SAA levels were significantly higher in the preterm group (22 mg/L) compared to the term group (7 mg/L) (p<0.001). The ROC curve analysis yielded a moderate predictive value of SAA for preterm birth, with the area under the ROC curve (AUC) being 0.690. The threshold at 15 mg/L was the best cut-off value, achieving a sensitivity of 89% and a specificity of 85%. Elevated SAA levels were associated with a 27.89-fold increased risk of preterm delivery. Further, after adjusting for maternal age, medical conditions during pregnancy, and prior preterm birth, elevated SAA remained a significant predictor of preterm birth (adjusted odds ratio (aOR)=28.966; p=0.001).

Conclusion: Maternal SAA proved to be a strong independent risk factor for preterm birth. This biomarker further narrows the population of pregnant women at higher risk of preterm delivery and opens new perspectives for its clinical role in preterm birth prevention.

## Introduction

Preterm birth, defined as delivery prior to 37 weeks of gestation, continues to pose a substantial public health concern globally, representing as the leading cause of newborn morbidity and death [1]. Notwithstanding improvements in perinatal care, preterm birth rates have not markedly declined, especially in low- and middle-income nations, where healthcare resources are frequently limited [2]. Worldwide, the average preterm birth rate is around 11%, with 15 million preterm births per year. Rates vary from 5% in certain European nations to as high as 18% in other African countries [2].

The pathophysiology of preterm birth is multifactorial, involving maternal, fetal, and environmental factors. Maternal inflammation is increasingly recognized as a critical factor in the pathogenesis of preterm birth. Pro-inflammatory cytokines and acute-phase reactants, such as C-reactive protein (CRP), interleukin-6 (IL-6), and tumor necrosis factor-alpha (TNF-α), are known to mediate processes that weaken fetal membranes and stimulate uterine contractions, ultimately leading to preterm delivery [3,4]. 

Serum amyloid A (SAA), an acute-phase protein, is primarily synthesized by the liver in reaction to inflammation and tissue damage. SAA has been extensively studied in various inflammatory diseases and is known to play a role in immune modulation and tissue remodeling [5]. This background suggests a potential for SAA to serve as a sensitive biomarker for other inflammatory conditions, including those associated with preterm birth. However, its potential role as a biomarker for predicting preterm birth has not been fully explored. 

This study aims to determine the predictive accuracy of maternal SAA levels for preterm birth in a Romanian cohort. By establishing a quantifiable biomarker, our research seeks to enhance early intervention strategies and tailor prenatal care to women at an increased risk of preterm delivery. 

## Materials and methods

Study design

This research was conducted as a case-control study to evaluate the predictive role of maternal SAA in preterm delivery. The research was carried out at the Department of Obstetrics and Gynecology, Timisoara Municipal Emergency Clinical Hospital. The study was conducted over a period spanning from April 2023 to March 2024.

Ethical approval for the study was obtained from the Ethics Committee of the Timisoara Municipal Emergency Clinical Hospital (approval number: E-1828/31.03.2023), and written informed consent was obtained from all participants before inclusion in the study.

Participants

The participants included pregnant women who were admitted to the Department of Obstetrics and Gynecology, Timisoara Municipal Emergency Clinical Hospital, exclusively for delivery while in spontaneous labor. A total of 136 pregnant women were enrolled in the study, of which 70 women who gave birth before 37 weeks of gestation were categorized into the case group (preterm birth group) and 66 women who gave birth at or after 37 weeks of gestation were categorized into the control group (term birth group). Inclusion criteria were singleton pregnancies and maternal age between 18 and 40 years. Exclusion criteria included pregnancies complicated by multiple gestations, congenital fetal anomalies, and maternal infections or systemic inflammatory diseases unrelated to pregnancy.

Data collection

Detailed information on participants' demographic characteristics, obstetric history, and medical conditions during pregnancy was collected from medical records and through direct interviews using a structured questionnaire. The primary outcome of interest was preterm birth, defined as birth before 37 weeks of gestation. Maternal SAA levels were measured at the time of admission for delivery. Maternal SAA levels were quantitatively assessed using the nephelometry method (NEPH). The measurements were performed using the Atellica® NEPH 630 System (Siemens Healthineers, Erlangen, Germany). 

Bias

To mitigate selection bias, participants were consecutively enrolled as they presented, at the Department of Obstetrics and Gynecology, Timisoara Municipal Emergency Clinical Hospital, in labor for delivery. Inclusion criteria were strictly adhered to, ensuring a representative sample of the target population. Additionally, our control group was matched based on key demographic and clinical parameters to the case group, using the nearest neighbor matching method. 

To prevent information bias, data collection was standardized across participants. All data were collected by trained medical personnel using a structured questionnaire. Maternal SAA levels were measured in a centralized laboratory with quality control measures in place to maintain assay consistency and reliability.

Furthermore, we used multivariate logistic regression analysis to adjust for potential confounders identified in the study. This statistical method controlled for variables such as maternal age, history of preterm delivery, and the presence of medical conditions during pregnancy.

Finally, to avoid sampling bias, sampling was conducted throughout the year to avoid seasonal variations in birth rates and potential related factors that could influence the outcomes. The extensive range of gestational ages and the comprehensive demographic profile help mitigate any bias that could arise from a more narrowly defined sample.

Statistical analysis

Data were analyzed using IBM SPSS Statistics for Windows, V. 25.0 (IBM Corp., Armonk, NY, USA). Continuous variables were expressed as median (interquartile range (IQR)) and compared between groups using the Mann-Whitney U test. Categorical variables were presented as frequencies (percentages) and compared using Fisher's exact test.

The predictive value of SAA for preterm delivery was assessed using receiver operating characteristic (ROC) curve analysis. The Youden index was used to determine the cut-off value that maximizes the sum of sensitivity and specificity.

Univariate and multivariate logistic regression analyses were performed to assess the association between SAA levels and preterm birth.

## Results

Baseline characteristics

A total of 136 patients were enrolled in the study, with 70 (51.5%) patients who gave birth preterm (preterm birth group) and 66 (48.5%) patients who gave birth at term (term birth group). The median age of patients was similar in both groups, with a median of 30 years in both preterm and term groups (p=0.19). A significant difference was observed in the history of previous preterm births, with 14 (21.2%) of the preterm group having a previous history, compared with only six (8.6%) in the term group (p=0.03). Although the presence of medical conditions during pregnancy was more common in the preterm group, 14 (21.2%), compared with the term group, eight (11.4%), this difference did not reach statistical significance (p=0.09) (Table 1).

**Table 1:** Characteristics of 136 patients included in this study Continuous variables are presented as median (interquartile range) and were analyzed using the Mann-Whitney U test. The U statistic is reported for these continuous variables. Categorical variables are presented as count (percentage) and were analyzed using Fisher's exact test. The odds ratio is reported as the test statistic to provide a measure of effect size.

Characteristics	Preterm birth group (n=70)	Term birth group (n=66)	P-value	Statistic
Maternal age	30 (8)	30 (9)	0.19	U=2014.00
Preterm birth history	14 (21.2%)	6 (8.6%)	0.03	OR=4.327
Medical conditions during pregnancy	14 (21.2%)	8 (11.4%)	0.09	OR=2.398
Gestational age at birth	31 (6)	39 (2)	<0.001	U=66.00

Amyloid A as a predictor in preterm birth

**Figure 1:**
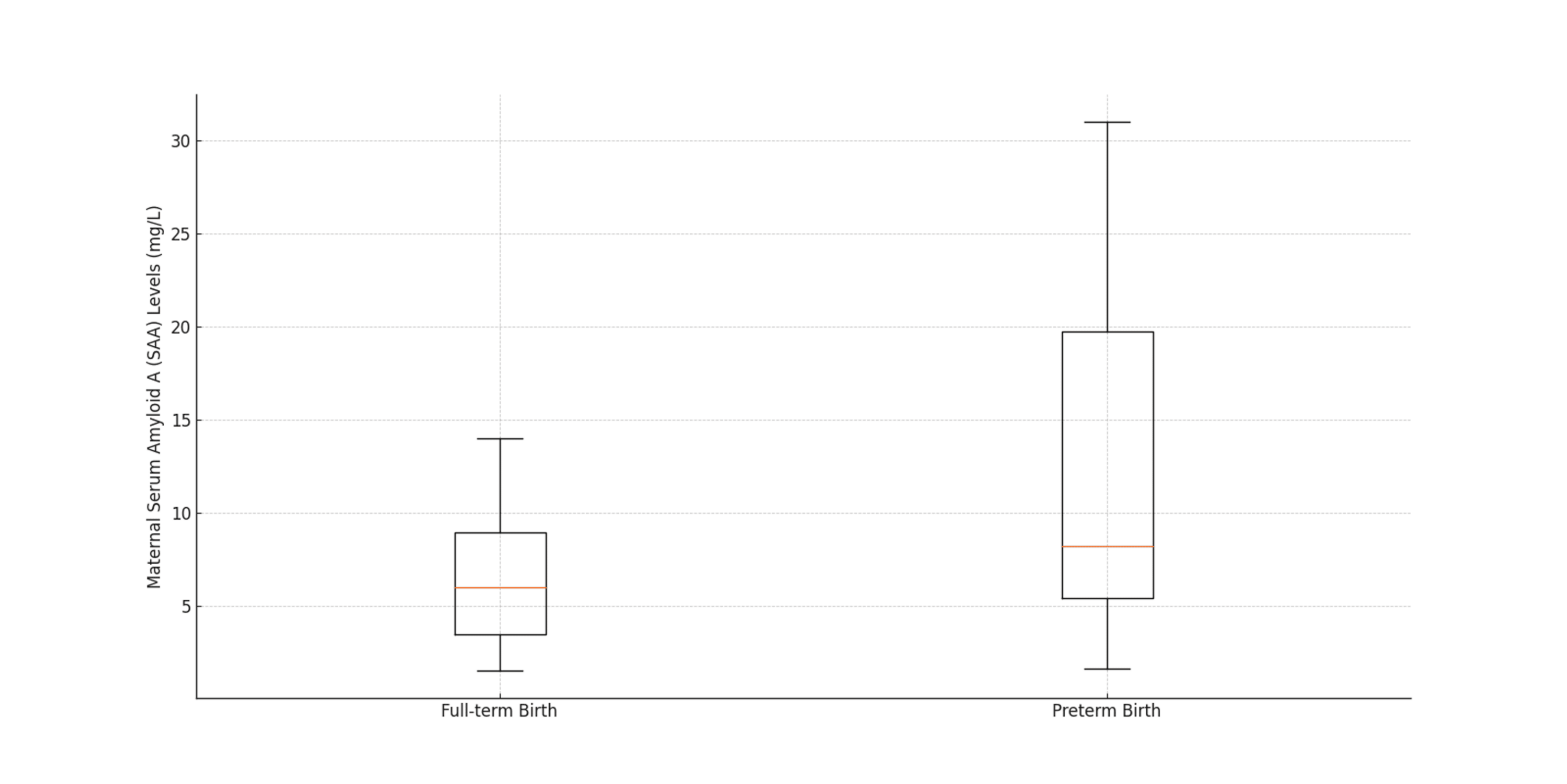
Distribution of maternal SAA levels in preterm and term birth groups SAA: serum amyloid A

Maternal SAA levels were higher in the preterm birth group, with a median of 8.2 mg/L (IQR 14.35) compared to 6 mg/L (IQR 5.45) in the term birth group; this difference was statistically significant (p<0.001) (Figure 1).

**Figure 2:**
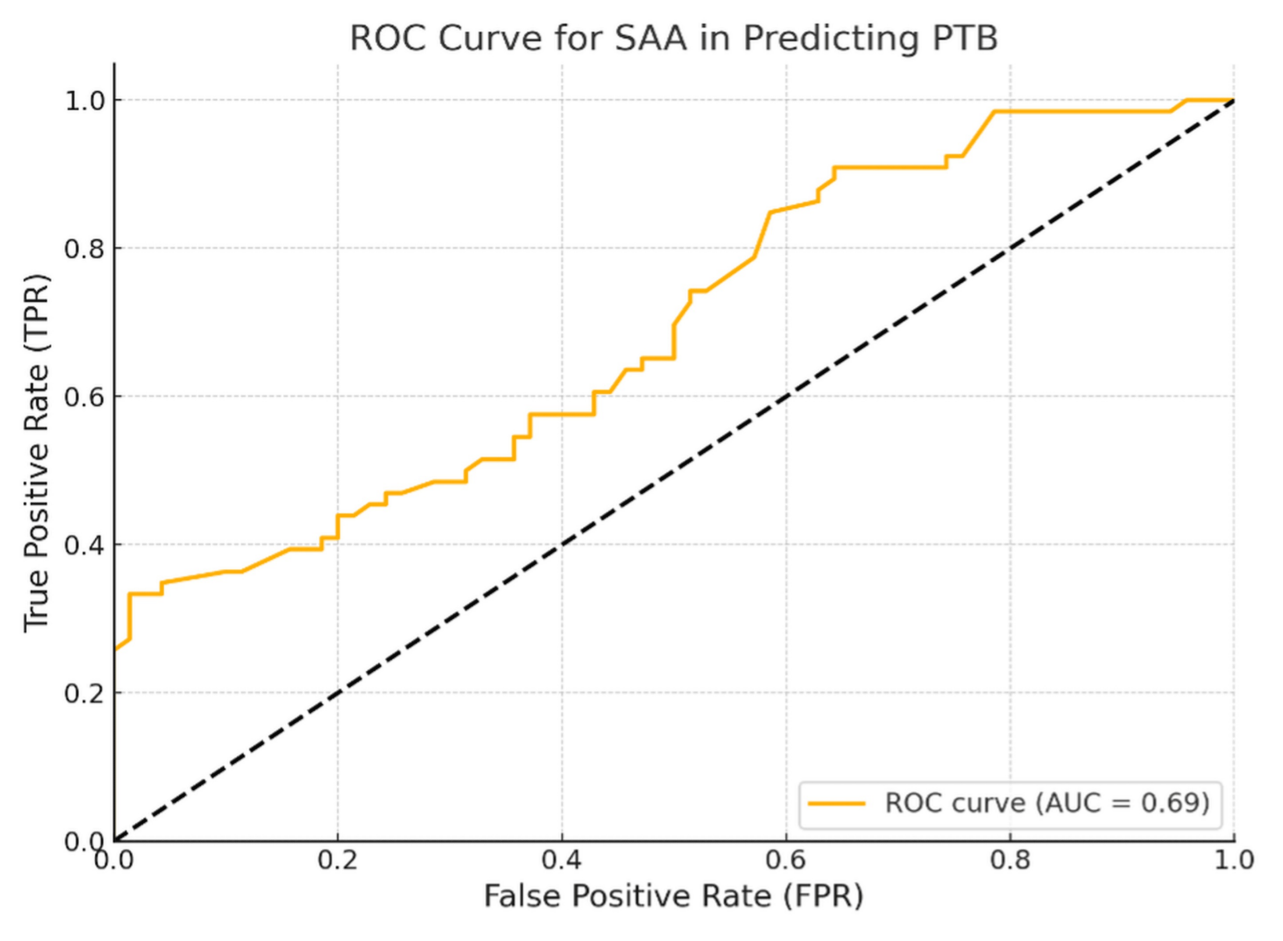
ROC curve for SAA predicting PTB ROC: receiver operating characteristic; SAA: serum amyloid A; PTB: preterm birth; AUC: area under the ROC curve

An ROC curve analysis was conducted to evaluate the predictive significance of maternal SAA in preterm delivery. The area under the ROC curve (AUC) for SAA was 0.690 (95% CI: 0.600-0.780; p<0.001) (Figure 2).

The AUC was 0.690, indicating that there was a good predictive value for SAA in identifying cases at risk of preterm birth. Using Youden's J statistic, the optimal cut-off value for SAA was found to be 15 mg/L with a sensitivity of 98.5% (Table 2).

**Table 2:** ROC curve analysis for predictive value of SAA in PTB *According to Youden's J statistic ROC: receiver operating characteristic; SAA: serum amyloid A; AUC: area under the ROC curve; FPR: false positive rate; TPR: true positive rate; PTB: preterm birth

AUC	Cut-off*	FPR	TPR	Sensitivity	Specificity
0.690	15 mg/L	0.014	0.33	33.3%	98.5%

**Figure 3:**
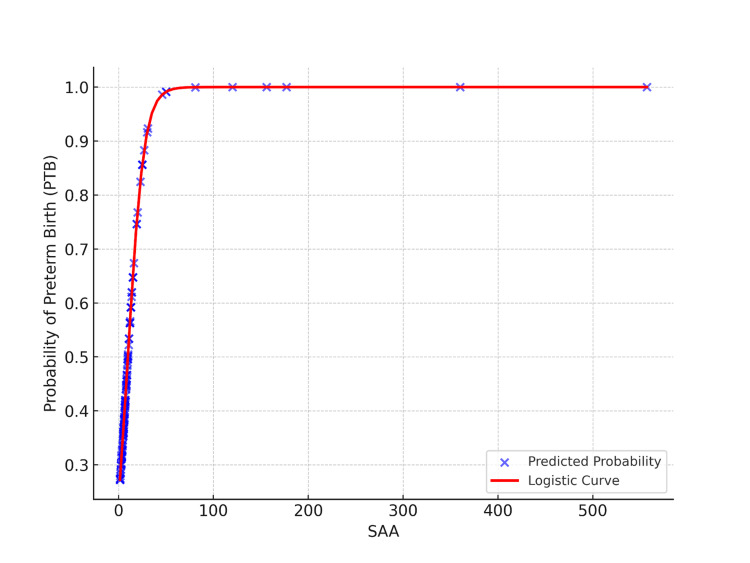
Regression analysis curve illustrating the predictive capacity of SAA for PTB SAA: serum amyloid A; PTB: preterm birth

Figure 3 depicts the multivariate logistic regression analysis curve for maternal SAA levels and their association with the risk of preterm birth.

Table 3 provides the results of the univariate logistic regression analysis examining the association between maternal SAA levels and the risk of preterm birth. The analysis reveals that each unit increase in SAA is associated with a 1.125-fold increase in the odds of preterm birth (odds ratio (OR)=1.125; 95% CI: 1.047-1.209; p=0.001).

**Table 3:** Logistic regression analysis of SAA and risk of PTB P-values were calculated using univariate and multivariate logistic regression. The first univariate logistic regression model used SAA as a continuous variable to measure the effect of each unit of SAA growth. The second univariate logistic regression model considered SAA as a categorical variable to investigate the impact of exceeding a threshold of 15 mg/L (cut-off value determined by ROC curve analysis). SAA: serum amyloid A; PTB: preterm birth; B: logistic regression coefficient; SE: standard error; OR: odds ratio; CI: confidence interval

Variable	B	SE	OR	P-value	95% CI	
					Lower	Upper
Univariate logistic regression						
SAA	0.117	0.037	1.125	0.001	1.047	1.209
Univariate logistic regression						
SAA >15 mg/L	3.328	1.043	27.894	0.001	3.610	215.54
Multivariate logistic regression						
SAA >15 mg/L	3.366	1.052	28.966	0.001	3.684	227.719
Maternal age	-0.053	0.033	0.948	0.11	0.888	1.012
History of PTB	1.359	0.576	3.892	0.01	1.258	12.043
Medical conditions during pregnancy	0.991	0.529	2.695	0.06	0.956	7.593

Furthermore, binomial logistic regression was used to assess the risk of preterm birth when the SAA level is above 15 mg/L (the cut-off value determined by ROC curve analysis). An SAA level above 15 mg/L is associated with a 27.89-fold increase in the risk of preterm birth compared with values below 15 mg/L, with a p-value of 0.001 (Table 3). 

A multivariate logistic regression analysis was performed to assess whether SAA levels >15 mg/L, along with other clinical factors, were predictive of preterm delivery. The results indicated that SAA >15 mg/L remained an independent predictor of preterm birth (p=0.001; adjusted OR (aOR)=28.9). In addition, in this model, preterm birth in the antecedents was a significant predictor of preterm birth (p=0.017; aOR=3.637; 95% CI: 1.257-10.521) (Table 3).

## Discussion

Our study demonstrated that elevated levels of SAA in pregnant women are associated with a significantly increased risk of preterm delivery. SAA values above the threshold of 15 mg/L were identified as an independent predictor for preterm delivery, with a 27.89-fold increased risk compared to values below this threshold. ROC curve analysis showed a moderate predictive value of SAA, with an AUC of 0.690, suggesting that SAA could be a useful inflammatory marker in assessing the risk of preterm birth.

Our findings are consistent with the existing literature emphasizing the role of inflammation and acute-phase proteins in the pathogenesis of preterm birth. For example, previous studies have shown that inflammatory proteins, such as CRP and IL-6, contribute to weakening fetal membranes and stimulate uterine contractions [6-10].

Inflammation in the feto-maternal environment is an important trigger for preterm delivery, and inflammatory processes in the amniotic cavity are frequently associated with premature induction of labor. Inflammation contributes to preterm birth primarily through two mechanisms: bacterial infections that invade the amniotic cavity and induce a severe inflammatory response and inflammation triggered by the direct presence of an infectious agent. Chorioamnionitis, an inflammation of the amniotic membrane, is commonly associated with intrauterine infections and induces the production of pro-inflammatory cytokines, such as IL-6 and interleukin-1 beta (IL-1β), which stimulate uterine contractions and contribute to the weakening of the fetal membranes [11]. On the other hand, sterile inflammation is driven by the release of endogenous molecules, such as high mobility group box 1 (HMGB1) proteins, IL-1α, IL-33, heat shock protein, and S100 protein, which are released in response to cell damage and can trigger significant inflammatory responses, including the activation of immunocompetent cells in the placenta [12].

SAA, an acute-phase reactant, has been implicated in various systemic inflammatory conditions and has been suggested as a potential biomarker for pregnancy-related complications, including preterm birth [13]. Ibrahim et al. suggested a more important role of inflammatory biomarkers in predicting preterm birth with an AUC of 0.972, compared to 0.690 in our study [14].

While our study provides valuable insights into the predictive accuracy of maternal SAA for preterm birth, several limitations should be considered. The study was conducted at a single tertiary care center, which may limit the generalizability of the findings to other settings or populations with different demographic and socioeconomic characteristics. While efforts were made to adjust for known confounders, there may be other unmeasured variables such as genetic factors, nutritional status, or undisclosed medical conditions that could influence maternal SAA levels and the risk of preterm birth. Although additional tests were conducted to exclude other sources of inflammation, it is possible that some subclinical inflammatory processes were not detected, which could confound the association between SAA levels and preterm birth. Finally, despite standardization, variations in assay performance and calibration could introduce measurement errors, potentially affecting the consistency of SAA-level determinations. 

## Conclusions

The study highlights that elevated maternal SAA levels are significantly associated with an increased risk of preterm birth, suggesting that SAA could serve as a useful biomarker for the early identification of high-risk pregnancies. With a threshold of 15 mg/L, elevated SAA levels were linked to a substantial increase in the likelihood of preterm delivery, underscoring the potential of SAA in refining prenatal risk assessment. By identifying women with heightened SAA levels, healthcare providers may be better equipped to monitor and manage those at risk for preterm birth, potentially implementing targeted interventions to improve maternal and neonatal outcomes. This study reinforces the value of inflammatory markers like SAA in understanding and predicting preterm birth, advocating for further exploration of its clinical application in prenatal care settings.
